# Editorial: Entrepreneurial psychological capital and emotional intelligence

**DOI:** 10.3389/fpsyg.2026.1919283

**Published:** 2026-08-06

**Authors:** Clara Margaça, Marián Cvirik, Helena Knörr

**Affiliations:** 1 Department of Social Psychology and Anthropology, University of Salamanca, Salamanca, Spain; 2 Faculty of Commerce, Bratislava University of Economics and Business, Bratislava, Slovakia; 3 Department of Management, Point Park University, Pittsburgh, PA, United States

**Keywords:** emotional intelligence, entrepreneurship, positive psychology, psychological capital, resilience, self-efficacy

Entrepreneurship is increasingly recognized as a psychological process that goes beyond recognizing opportunities, innovating, and creating ventures. In an era characterized by rapid technological change, economic uncertainty, and increasingly complex social challenges, entrepreneurs, employees, leaders, and students are expected not only to possess technical knowledge and professional skills but also to demonstrate the psychological resources necessary to adapt, persist, and thrive in dynamic environments. Consequently, the study of Entrepreneurial Psychological Capital (PsyCap) and Emotional Intelligence (EI) has attracted increasing academic attention as researchers seek to better understand the cognitive, emotional, and relational mechanisms that underpin entrepreneurial behavior, proactive attitudes at work, and sustainable individual development.

PsyCap, conceived as a higher-order construct encompassing hope, self-efficacy, resilience, and optimism, represents a malleable psychological resource that enhances individuals' ability to overcome adversity, pursue challenging goals, and maintain motivation under conditions of uncertainty (Luthans et al., 2007; Luthans and Youssef-Morgan, 2017). Unlike relatively stable personality traits, PsyCap can be intentionally developed through educational experiences, leadership practices, and organizational interventions, making it particularly relevant to entrepreneurship (Margaça et al., 2023), where uncertainty, ambiguity, and continuous adaptation are inherent characteristics. Emotional Intelligence, the ability to perceive, understand, regulate, and use emotions effectively (Mayer et al., 2016), has emerged as a fundamental capacity for managing interpersonal relationships, navigating complex social environments, making sound decisions, and maintaining psychological wellbeing. Recent meta-analysis evidence further demonstrates that emotional intelligence contributes positively to job performance, leadership effectiveness, organizational citizenship behaviors, and resilience in various organizational contexts (Miao et al., 2017).

These developments reflect the broader influence of Positive Organizational Psychology, which has progressively shifted attention from deficit-focused approaches to understanding the positive psychological resources that enable individuals and

organizations to thrive (Donaldson and Ko, 2010; Luthans and Youssef-Morgan, 2017). In the context of entrepreneurship research, this perspective has encouraged researchers to move beyond explanations focused exclusively on personality traits or economic incentives, recognizing instead that entrepreneurial capacity is shaped by the dynamic interaction between individual psychological resources and the educational, organizational, and social environments in which individuals operate (Obschonka et al., 2012; Obschonka and Stuetzer, 2017). Entrepreneurship is therefore increasingly seen as a developmental process in which adaptive capabilities, emotional skills, and supportive contexts jointly influence individuals' ability to recognize opportunities, cope with setbacks, engage in proactive behaviors, and generate value for organizations and society.

This Research Topic aimed to deepen the understanding of the interaction between Entrepreneurial PsyCap and EI, bringing together contributions that explore how positive psychological resources influence human behavior in educational and organizational contexts. Although the published articles explore diverse populations, including university students, employees, language learners, and organizational leaders, and examine different psychological mechanisms, they collectively address a common question: how do internal psychological resources interact with contextual conditions to promote adaptive, proactive, and entrepreneurial behaviors? Instead of considering entrepreneurship solely as the act of creating new ventures, the Research Topic adopts a broader psychological perspective, in which entrepreneurial potential is understood as a constellation of attitudes, emotional competencies (e.g., Menon et al., 2023: Pathak and Muralidharan, 2024), values, and behavioral capabilities that can be cultivated throughout different phases of personal and professional development.

The five contributions consider that entrepreneurial PsyCap is neither an isolated individual attribute nor an innate characteristic possessed by a select group of entrepreneurs. Instead, studies consistently demonstrate that positive psychological resources develop through continuous interactions between individuals and their educational and organizational environments. In doing so, this Research Topic expands contemporary discussions on entrepreneurial development, highlighting the complementary roles of leadership, learning environments, personal values, emotional processes, and psychological capital in shaping behaviors that ultimately support innovation, employability, organizational effectiveness, and entrepreneurial intent. Although these contributions address different educational and organizational contexts, they converge around a common proposition: positive psychological resources act as mechanisms by which individuals transform challenges into adaptive behaviors. Instead of portraying entrepreneurial psychological capital as a fixed personal attribute, the studies collectively demonstrate that it develops through the interaction between individual capabilities, interpersonal relationships, and contextual influences.

The mediating role of psychological resources is particularly evident in the study by Chen, Alruwaili et al., which extends the application of PsyCap to foreign language teaching. Using a large sample of Chinese learners of English as a foreign language, the authors demonstrate that PsyCap partially mediates the relationship between language-specific perseverance and the willingness to communicate, highlighting hope, optimism, resilience, and self-efficacy as critical resources that enable individuals to translate perseverance into adaptive behavioral outcomes. Instead of being limited to organizational contexts, these findings reinforce the broader applicability of PsyCap as a developmental resource capable of improving performance in diverse learning environments.

Liu et al. show that employee hope mediates the relationship between perceived leader competence and feedback-seeking behavior for self-improvement, while Leader-Member Exchange strengthens this indirect effect, demonstrating that supportive interpersonal relationships amplify the benefits of positive psychological resources. Similarly, Chen, Zhao et al. demonstrate that leader humility promotes proactive employee behavior through sequential increases in positive mood and affective commitment, and role breadth self-efficacy reinforces this process. Both studies illustrate that leadership influences behavior not simply through authority or formal structures, but by creating emotional and psychological conditions that foster trust, learning, initiative, and proactive action. Although emotional intelligence is not explicitly measured, both studies underscore the importance of emotionally competent leadership in cultivating the psychological resources that underpin adaptive behavior in the workplace.

Ke et al. demonstrate that cognitive-emotional processes link eudaimonic life values to work stress, while educational background shapes the strength of these relationships, suggesting that career adaptability is influenced by both personal values and contextual factors. Similarly, Bagis et al. argue that entrepreneurial education alone is insufficient to strengthen entrepreneurial intent among business students. Instead, character strengths, universal values, and teacher skills emerge as the main mechanisms by which entrepreneurial intentions are developed, highlighting that entrepreneurial education is most effective when it fosters psychological growth alongside knowledge acquisition.

Hope, perseverance, self-efficacy, positive mood, affective commitment, character strengths, and values consistently act as catalysts that enable individuals to respond constructively to educational, organizational, and career-related challenges. Simultaneously, leadership behaviors, learning environments, and supportive interpersonal relationships create the contextual conditions under which these resources can flourish. Consequently, this Research Topic deepens the understanding of entrepreneurial psychological capital, demonstrating that entrepreneurial capacity is not merely a product of individual characteristics, but rather emerges from the continuous interaction between psychological resources and the environments in which individuals learn, work, and develop.

The studies included in this Research Topic offer valuable insights into the psychological foundations of entrepreneurial behavior, as well as highlighting several promising avenues for future research. First, the predominantly cross-sectional designs of the studies limit our understanding of how entrepreneurial psychological capital and emotional intelligence evolve over time. Longitudinal studies would be valuable for examining the development of these psychological resources at different career stages and entrepreneurial trajectories. Similarly, intervention-based research could explore the effectiveness of educational programs, leadership development initiatives, and organizational practices specifically designed to strengthen psychological capital and emotional skills. Secondly, the Research Topic illustrates the importance of contextual influences, including leadership, educational environments, and interpersonal relationships. Future studies could explore these multi-level dynamics, analyzing how institutional cultures, organizational climates, and national contexts interact with individual psychological resources to shape entrepreneurial outcomes. Cross-cultural comparative research would also contribute to assessing the generalizability of current findings, particularly since most contributions in this Research Topic have been conducted in Asian educational and organizational contexts. Finally, entrepreneurship is increasingly shaped by digital transformation, artificial intelligence, and sustainability challenges, creating new demands for adaptability, emotional regulation, ethical decision-making, and lifelong learning. Future research should analyze how Entrepreneurial Psychological Capital and Emotional Intelligence support entrepreneurial action in technology-driven environments and contribute to addressing broader social challenges through sustainable and socially responsible entrepreneurship.

The contributions of this Research Topic reinforce the growing recognition that entrepreneurship is a human process, grounded not only in technical knowledge and skills, but also in positive psychological resources that enable individuals to recognize opportunities, cope with uncertainty, build meaningful relationships, and persevere in the face of adversity.
